# Acute oral administration of low doses of methylphenidate targets calretinin neurons in the rat septal area

**DOI:** 10.3389/fnana.2015.00033

**Published:** 2015-03-23

**Authors:** Álvaro García-Avilés, Héctor Albert-Gascó, Isabel Arnal-Vicente, Ebtisam Elhajj, Julio Sanjuan-Arias, Ana María Sanchez-Perez, Francisco Olucha-Bordonau

**Affiliations:** ^1^Departamento de Medicina, Facultad de Ciencias de la Salud, Universitat Jaume ICastellón, Spain; ^2^CIBERSAM, INCLIVA, Unidad de Psiquiatría, Departamento Medicina, Facultad Medicina, Universitat de ValenciaValencia, Spain; ^3^Faculty of Science, University of AlexandriaAlexandria, Egypt

**Keywords:** ADHD, methylphenidate, catecholamines, calcium binding proteins, theta rhythm

## Abstract

Methylphenidate (MPD) is a commonly administered drug to treat children suffering from attention deficit hyperactivity disorder (ADHD). Alterations in septal driven hippocampal theta rhythm may underlie attention deficits observed in these patients. Amongst others, the septo-hippocampal connections have long been acknowledged to be important in preserving hippocampal function. Thus, we wanted to ascertain if MPD administration, which improves attention in patients, could affect septal areas connecting with hippocampus. We used low and orally administered MPD doses (1.3, 2.7 and 5 mg/Kg) to rats what mimics the dosage range in humans. In our model, we observed no effect when using 1.3 mg/Kg MPD; whereas 2.7 and 5 mg/Kg induced a significant increase in c-fos expression specifically in the medial septum (MS), an area intimately connected to the hippocampus. We analyzed dopaminergic areas such as nucleus accumbens and striatum, and found that only 5 mg/Kg induced c-fos levels increase. In these areas tyrosine hydroxylase correlated well with c-fos staining, whereas in the MS the sparse tyrosine hydroxylase fibers did not overlap with c-fos positive neurons. Double immunofluorescence of c-fos with neuronal markers in the septal area revealed that co-localization with choline acethyl transferase, parvalbumin, and calbindin with c-fos did not change with MPD treatment; whereas, calretinin and c-fos double labeled neurons increased after MPD administration. Altogether, these results suggest that low and acute doses of methylphenidate primary target specific populations of caltretinin medial septal neurons.

## Introduction

Attention deficit hyperactivity disorder (ADHD) is a developmental disorder affecting an estimate of 3–5% of children worldwide. In the 60–80% of cases, symptoms persist into adolescence and adulthood (Polanczyk et al., 2007). The aetiology of this disorder is not fully understood, but current belief points to alterations in the dopaminergic system underling the onset of the disease. For instance, mutations and polymorphisms in the dopamine transporter (DAT) have been associated with ADHD (Miller and Madras, 2002). Other studies show an increase in DAT density up to 70% in ADHD patients compared to healthy volunteers (Dougherty et al., 1999). Furthermore, reduced dopamine activity has been reported in striatal areas of ADHD patients (Volkow et al., 2007). Also, alterations in the dopamine receptor D1 mediated neurotransmission in specific brain regions have been described in animal models of ADHD, such as the spontaneously hypertensive rats (SHR; Russell et al., 1995; Ohno et al., 2012).

Symptoms observed in ADHD patients include inattention, hyperactivity and impulsivity (DSM-5) (Biederman and Faraone, 2005; Biederman, 2006). In these patients, attention deficits positively correlate with impaired executive functions as for example working memory (Takeuchi et al., 2013). In addition, in SHR rats, hyperactivity has been strongly associated with deficits in working memory, response inhibition and motor timing (Kuntsi et al., 2006).

It is well known, that hippocampal theta rhythm underlie working memory requiring optimal attention and awareness processes (Hasselmo, 2005). Therefore, a number of studies have been devoted to understand the mechanisms modulating it. Several brain areas have been demonstrated to have different roles as generators or modulators of the hippocampal rhythm. Behavioral studies have shown that the MS regulate tasks that demand attention (Brandner and Schenk, 1998; Walsh et al., 1998; Ma et al., 2002). Moreover, the integrity of the entire medial and lateral septum-hippocampal network has been shown to be critical for theta rhythmogenesis (Vertes and Kocsis, 1997; Hasselmo, 2005; Nerad and McNaughton, 2006). In particular, it has been established that predominantly GABAergic and cholinergic neurons in the MS function as pacemaker for the hippocampal theta rhythm (Lee et al., 1994; Vertes and Kocsis, 1997; Apartis et al., 1998; Wang, 2002; Yoder and Pang, 2005).

The current treatment for ADHD is methylphenidate (MPD; Abikoff et al., 2004). MPD inhibits dopamine re-uptake by binding to the extracellular domain of the DAT and, as a result, increasing dopamine (DA) and noradrenaline (NE) concentration in the synaptic cleft (Zetterström et al., 1988; Volkow et al., 1995). Studies using DAT knockout mice demonstrated that dopaminergic and norepinephric transmission mediates MPD induction of c-fos expression in a number of brain areas (Trinh et al., 2003). Catecholamine projections to septal area have been reported (Sánchez-Camacho et al., 2003), moreover injection of D1 receptor agonists in the MS have been demonstrated to induce acetylcholine neurotransmitter increase in hippocampus (Zarrindast et al., 2012). Furthermore, D1 and D5 activation enhances the firing rates of medial septal neurons that provides a state with higher probability for hippocampal theta occurrence (Fitch et al., 2006), Thus we hypothesized that MPD could exerts its effects improving attention processes via modulating the septo-hippocampal circuitry. To our knowledge, up to date no detailed studies of MPD effects on neuronal activity within septal regions have been carried out. We measured neuronal activity by analyzing c-fos expression, which has long been considered as an inducible marker of neuronal activity, particularly after acute stimulation (Curran and Morgan, 1995; Kovács, 2008). Moreover, we wanted to compare our findings with previous reports on MPD activity that had used c-fos expression as measure of brain activation (Trinh et al., 2003). In our model, we observed that low doses of MPD can induce specifically c-fos activation in calretinin neuronal populations within the septum, which may have important consequences on septo-hippocampal connections, modulating hippocampal function.

## Materials and Methods

### Animals and Storage Conditions

Procedures were in line with directive 86/609/EEC of the European Community on the protection of animals used for experimental and other scientific purposes and approved by the ethic committee of the University of Valencia. A total of 21 adult female Sprague-Dawley rats between 200–300 g were used. All animals were maintained on a 12 h light cycle provided with food and water ad libitum. The animals were randomly divided into three differential groups according to MPD treatment i.e., “Control”, “MPD 1.3”, “MPD 2.7” and MPD “5”.

### Drug Treatment

Methylphenidate (MPD, Rubifem, Laboratories Rubió, S.A, Barcelona-Spain) was administered orally, mixed with orange flavored jelly. Rats were habituated to jelly taste for three days prior to drug administration on the fourth day. The control group received jelly alone; whereas the MPD 1.3; MPD 2.7 and MPD 5 groups received 1.3; 2.7 and 5 mg/Kg of MPD respectively. The equations should be inserted in editable format from the equation editor.

### Perfusion and Fixation

Animals were euthanized with an i.p. injection of sodium pentobarbital (120 mg/Kg Eutanax, Fatro, Barcelona, Spain) 90 min after either jelly or drug administration. Rats were transcardially-perfused with saline (0.9% NaCl, 250 ml) followed by fixative (4% paraformaldehyde in 0.1 M PB, pH 7.4) for 30 min (~600 ml). After perfusion, brains were removed from skull and immersed in the same fixative for 4 h at 4°C. After fixation, brains were cryoprotected in 30% sucrose in 0.01 M phosphate buffered saline (PBS) pH 7.4 for 3 days. Coronal sections (40 µm) were obtained using a freezing slide microtome (Leica SM2010R, Heidelberg, Germany). For each brain, 6 series of sections were collected in 30% sucrose in 0.01 M PBS. One of the series was directly used for c-fos immunohistochemistry and the rest were frozen at −40°C or used for double labeling.

### c-fos Immunocytochemistry and Double Labeling

Sections were rinsed 3 × 10 min in 0.05 M pH 8.0 Tris Buffer Saline (TBS). Afterwards, sections were transferred to a blocking solution containing 4% normal goat serum (NGS Jackson Immunoresearch, West Grove, PA, USA), 2% bovine serum albumin (BSA, (Sigma, St Louis Mo) and 0.1% Triton X-100 (Sigma, St Louis Mo) in TBS for 1 h. Sections were incubated in a medium containing 1:10000 rabbit anti-cfos (PC38 Anti-c-fos (Ab-5), EMD Millipore, Billerica, MA, USA) in the same blocking solution for 48 h at 4°C. After removing the first antibody with several washes in TBS, biotinylated secondary antibody (1:200 biotinylated donkey anti-rabbit; Cat No. 711-065-152, Jackson Immunoresearch, West Grove, PA, USA) was added to slides for 2 h. Sections were then rinsed and transferred to 1:50 ABC (Vectastain-Elite, Cat No. PK-6100; Vector Laboratories, Burlingame, CA, USA). After rinsing (2 × Tris-HCl) the immunolabeling was revealed as a black reaction product by immersing the sections in 0.025% DAB, 0.08% ammonium nickel sulfate, 0.0024% H_2_O_2_ in Tris HCl, pH 8.0. Prior to mounting on chrome-alum gelatine-coated slides, tissue was washed for at least 2 h in 0.01 M PBS. After air-dried, de-hydrated in graded ethanol, cleared with xylene, tissue was finally coverslipped with DPX (Sigma, St Louis Mo, USA).

Double labeling c-fos-TH was carried out following the same protocol as above but using sequentially antisera c-fos, then anti-sera against TH (1:1000 mouse anti-TH, T2928, Sigma, St Louis Mo, USA). After c-fos labeling was developed (obtaining black c-fos positive neurons), sections were rinsed overnight and the next day incubated with biotinylated secondary antibody against TH primary antibody (1:200 biotinylated goat anti-mouse; Cat No. 711-065-152, Jackson Immunoresearch, West Grove, PA, USA) for 2 h. The protocol was the same as the one used for c-fos, but removing the ammonium nickel sulphate in the final developing reaction, thus we detected TH positive fibers as a light brown color.

For double immunofluorescence, sections were processed as above, using 1:4000 rabbit anti c-fos, and either 1:5000 mouse anti parvalbumin (PV235, Swant, Marly Switzerland), 1:2500 mouse anti-calretinin (6B3, Swant) or 1:5000 mouse anti calbindin-28 kD (CB300, Swant, Marly Switzerland), and 1:500 anti ChAT (AB144, Chemicon, International, Inc. Temecula, USA) in 2% normal donkey serum (NDS Jackson Immunoresearch, West Grove, PA, USA), 2% BSA in TBS Tx100 for 48 h at 4°C. After rinsing, sections were incubated with 1:200 donkey anti rabbit-Cy3 (711-165-152, Jackson) and 1:200 donkey anti mouse (715-095-150, Jackson, Immunoresearch, West Grove, PA, USA) for the combination of c-fos and calcium binding proteins (PV, CB and CR) or 1:200 donkey anti rabbit-Cy3 (711-165-152, Jackson, Immunoresearch, West Grove, PA, USA) and 1:200 donkey anti goat (705-545-003, Jackson, Immunoresearch, West Grove, PA, USA) for the combination of c-fos and ChAT. The secondary fluorescent antibodies were incubated for 2 h. After incubation, the sections were rinsed in 0.01 M PBS and coverslipped in fluorsave (345789, Millipore, Darmstadt, Germany).

### Image Analysis and Neuron Quantification

Images were acquired using a Nikon Eclipse E600 (Nikon, Tokyo, Japan), equipped with a Nikon DMX-2000 camera connected to a PC with ACT-1 acquisition software (Nikon, Tokyo, Japan).

For c-fos quantification we used the 20x objective and measure c-fos activity as described (Perez-Villalba et al., 2005). Briefly, using Image J software, the background of the images was automatically removed with a rolling ball radius of 50.0 pixels, only labeled areas of more than 12 pixels were considered to be positive. Data was expressed as the ratio of c-fos positive area normalized to total area analyzed, according to the morphology of the nuclei examined (Table 1). All analyses were conducted by an observer blind to experimental conditions. We used c-fos positive area rather that number of c-fos positive neurons in order to minimized automated errors.

**Table 1:** **Septal nuclei c-fos levels at the indicated MPD doses**.

	Vehicle	MPD 1.3 mg/Kg	MPD 2.7 mg/Kg	MPD 5 mg/Kg
Areas	Mean ± SEM	Mean ±SEM	Mean ± SEM	Mean ± SEM
MS/VDB	0.86 ± 0.03	0.49 ± 0.08	1.64 ± 0.29 *** ###	1.32 ± 0.10 *##
HDB	1.00 ± 0.09	0.78 ± 0.16	0.96 ± 0.17	0.99 ± 0.07
LSI	1.00 ± 0.08	1.15 ± 0.11	1.57 ± 0.52	1.17 ± 0.08
LSD	1.00 ± 0.10	0.68 ± 0.21	0.96 ± 0.19	1.07 ± 0.07
LSV	1.00 ± 0.10	1.27 ± 0.12	1.23 ± 0.27	1.08 ± 0.08
TS	0.99 ± 0.20	1.08 ± 0.28	1.09 ± 0.16	1.13 ± 0.07
SFi	1.00 ± 0.10	0.83 ± 0.25	1.01 ± 0.41	1.00 ± 0.15
Striatum	1.07 ± 0.12	1.05 ± 0.31	1.12 ± 0.23	2.18 ± 0.29 **#
Accubens	1.00 ± 0.11	0.83 ± 0.18	1.53 ± 0.26	1.16 ± 0.11
MeA	1.00 ± 0.08	0.95 ± 0.20	1.10 ± 0.33	1.01 ± 0.13
BLA	1.06 ± 0.09	1.11 ± 0.16	1.17 ± 0.25	1.44 ± 0.22

The area of c-fos positive neurons was studied as indicated in the text. Controls and drug were normalized to the average value of controls. Data was analyzed by ANOVA followed by Bonferroni *post hoc* test. *p < 0.05, ** p < 0.01.

Analyses of differences in c-fos immunoreactivity were done using One-Way Analyses of Variance followed by *post hoc* analyses (Bonfferoni test ) with probability set at *α* < 0.05, using Graphpad Prism version 5 software.

Confocal immunofluoresence was imaged with a laser confocal scan unit TCS-SP2 equipped with argon and helio-neon laser beams attached to a Leica DMIRB inverted microscope (Leica Microsystems). Wavelengths for Cy3 excitation was 433 nm and for emission 560–618 nm; Alexa488-labeled antibody excitation was 488 nm and for emission was 510–570 nm. Serial 1 µm scans were obtained in the *Z*-plane and a maximal projection of 0.5 µm was generated with Leica confocal software (V2.61).

For double labeled neuron quantification, total number of CR, PV, CB and ChAT positive neurons were quantified in at least ten different photographs from septum taken with the 20x objective from at least three subjects. Double labeled neurons were counted blindly to the treatment (no drug or 5 mg/Kg MPD) and expressed as a percentage of total CR, PV, CB or ChAT positive cells.

## Results

This study aims to clarify the relationship between neural activity in septal areas (associated to attention and arousal mechanisms) and MPD treatment. Particularly, we were interested in doses and way of administration relevant to human applications. Our results emphasized the specificity of MPD treatment and the relevance of CR neurons within septal areas. Using c-fos as a marker, we found that MPD at low but clinically relevant doses induces CR positive neurons activity in specific septal nuclei controlling alertness and wakeful circuitries. These data suggest that CR septal neurons may be a first target of the drug. In addition, we examined areas with high TH staining, including striatum and nucleus accumbens.

### c-fos Expression in Septal Nuclei

**Figure 1:**
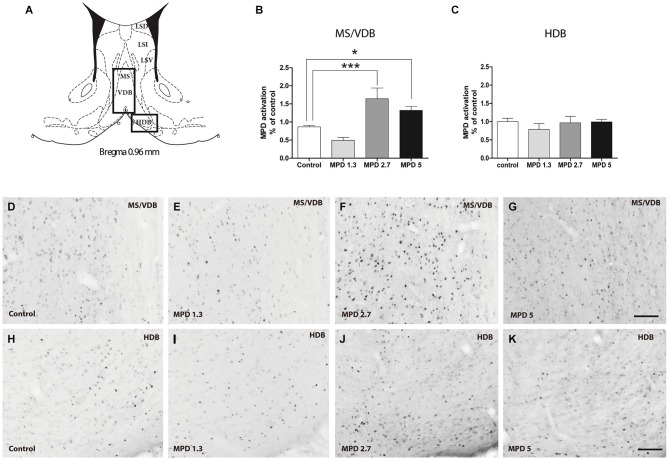
**c-fos expression augments with MPD treatment in MS/VDB, but not horizontal diagonal band (HDB)**. Map showing the location of the analyzed areas vertical medial septum (MS/VDB) and HBD **(A)**. Graphs showing the quantification of c-fos positive area expressed as the percentage of the total area analyzed and normalized to the average of control values in MS/VDB **(B)** and HDB **(C)**. Results were analyzed with one-way ANOVA, followed by Bonferroni *post hoc* test **p < 0.01). Representative images of c-fos staining in the MS/VDB vehicle or MPD treated at the indicated dosages **(D–G)**; representative images of c-fos neurons in the HBD from rats treated with vehicle or MPD at the indicated dosages **(H–K)**. Calibration bar 100 µm.

We examined septal nuclei following the cytoarchitectonic map proposed by Paxinos and Watson (Paxinos and Watson, 1996). We first analyzed the effect of MPD administration within the vertical medial septum (MS/VDB) and the horizontal diagonal band (HDB) corresponding to anterior septal nuclei, Bregma 0.96–0.48 nm (Figure 1A). One-way ANOVA analysis followed by Bonferroni post/hoc test revealed that MPD 2.7 and MPD 5 induced a significant increase in c-fos expression in MS/VDB, (*p* < 0.0001, *F*_(3,30)_ = 13.66) (Figure 1B) but not in the HDB (*p* = 0.6441, *F*_(3,31)_ = 0.5621) (Figure 1C) compared to MPD 1.3 and controls. Representative images of c-fos staining are shown for MS/VDB (Figures 1D–G) and for HDB (Figures 1H–K), at the indicated MPD doses. c-fos was distributed homogeneously throughout central and lateral aspects of the MS/VDB area.

We further studied the Lateral Septum (LS) (Bregma 0.96–0.48 nm), discriminating between dorsal (LSD) inter-mediate (LSI) and ventral parts (LSV). One-way ANOVA analysis of c-fos data showed no significant differences within LSD (*p* = 0.3205, *F*_(3,31)_ = 1.223), LSI (*p*= 0.6726, *F*_(3,31)_ = 0.5186) (not shown) and LSV (*p* = 0.5211, *F*_(3,32)_ = 0.7668). Representative images are illustrated in bottom panels for LSD and for LSV. Similarly, in the posterior septum, Triangularis Septalis (TS) and the Septofimbrial (SFi) we did not observe significant differences in c-fos expression; TS (*p* = 0.9679, *F*_(3,30)_ = 0.08468) and SFi (*p* = 0.9431, *F*_(3,32)_ = 0.1275). These data are summarized in Table 1.

### c-fos, Amygdala

Since amygdala is involved in regulating emotional processes and only intercalated nuclei displayed a higher concentration of TH positive fibers, c-fos activation in this area could be found as a negative control after MPD treatment. We analyzed two amygdala nuclei, medial amygdala (MeA) and basolateral amygdala (BLA), and, as expected, we found no significant differences (data summarized in Table 1).

### c-fos, Striatum and Nucleus Accumbens

**Figure 2:**
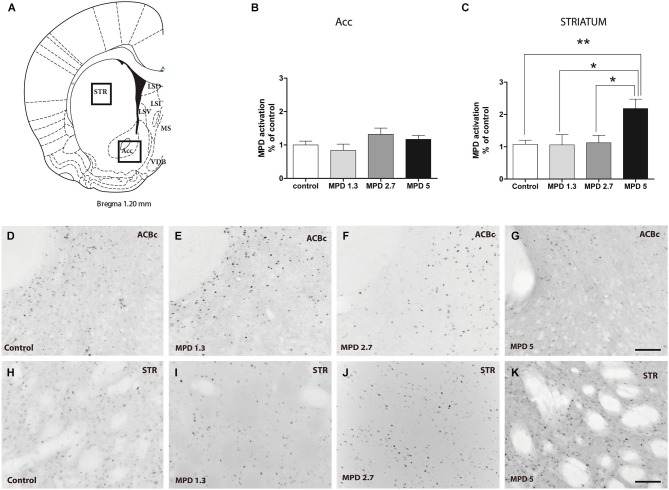
**MPD induces c-fos expression in Striatum but not nucleus accumbens**. Map showing the location of the analyzed areas **(A)**. Quantification of c-fos positive area represented as percentage of total area analyzed and normalized by average value of controls, accumbens shell **(B)** and striatum **(C)**. Results analyzed with one-way ANOVA, followed by Bonferroni *post hoc* test *p < 0.05. Representative images of c-fos staining in accumbens shell **(D–G)** and striatum **(H–K)** in vehicle and MPD treated rats at the indicated concentrations. Calibration bar 100 µm.

It is well accepted that MPD affects DA signaling, thus, we set out to confirm previous results in areas with high TH content as a control for MPD effect on neuronal activity. We examined nucleus accumbens and striatum, Bregma 1.20–0.96 nm (Figure 2A). Statistical analysis showed that MPD treatment did not induced significant differences in c-fos expression within the nucleus accumbens (Figure 2B) (*p* = 0.2328, *F*_(3,31)_ = 1.505), whereas it had a significant effect within the striatum (*p* = 0.0043, *F*_(3,30)_ = 5.406) (Figure 2C). Interestingly, we only could see this effect when using MPD 5 mg/Kg, but not at 2.7 mg/Kg or lower dosages. Representative pictures are shown for nucleus accumbens (Figures 2D–G) and striatum (Figures 2H–K).

### Double Labeling TH and c-fos

**Figure 3:**
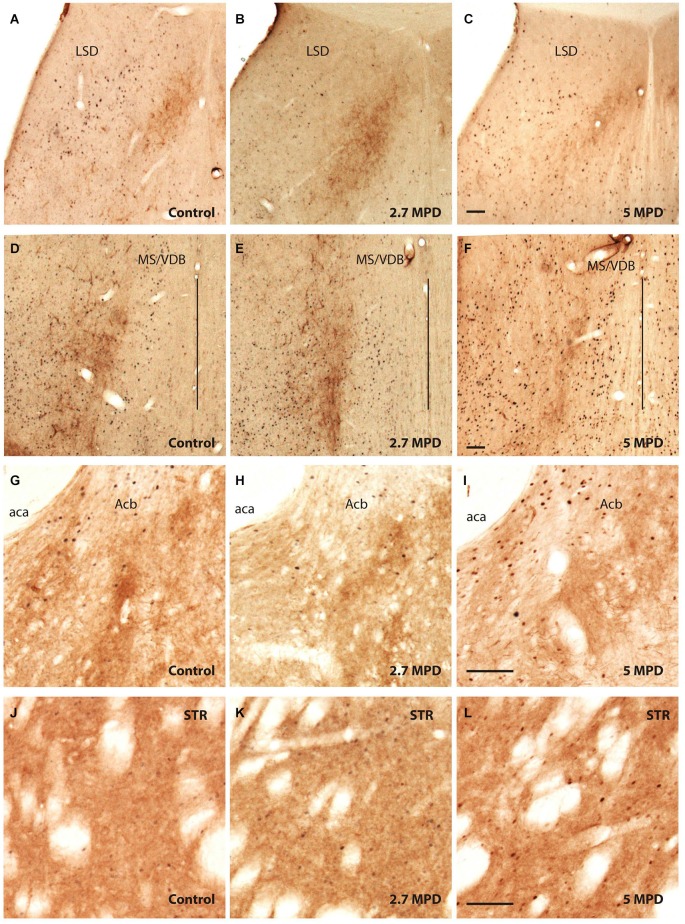
**Double immunocitochemistry TH-c-fos**. Representative images illustrating double TH and c-fos staining in the dorsal part of LS **(A–C)**; MS/VDB **(D–F)**; nuccleus accumbens **(G–I)** and striatum **(J–L)** in vehicle and MPD treated rats at the indicated dosages. The vertical line **(D–F)** represents the middle line of septum. Calibration bar 100 µm **(A–L)**.

Confirming previous data we observed that the LSD showed poor TH labeling (Figures 3A–C). On the other hand, TH staining showed an evident stripe of processes located lateral to the MS/VDB, but not in close proximity of MPD-induced c-fos neurons which are found more medial (control Figures 3D–F).

We show strong TH labeling in the Nucleus Accumbens (Figures 3G–I), were c-fos expression did not augment following MPD treatment. On the other hand, Striatum with high TH staining MPD induced c-fos expression at 5 mg/Kg (Figures 3J–L).

### Characterization of c-fos Positive Neurons in the Medial Septum

Although the different types of neurons may seem distributed without a distinctive pattern in the septal area, a rough model can be defined and corresponded to the prototype we have previously described. Briefly, it has been described that ChAT-positive neurons occupy lateral aspects of the MS/VDB and concentrate in a superficial band in the HDB. On the contrary, PV neurons populate mostly medial aspects of the MS/VDB, whereas CR and CB neurons concentrate dorsal and laterally in areas of the MS/VDB devoid of PV-positive neurons (Olucha-Bordonau et al., 2012).

**Figure 4:**
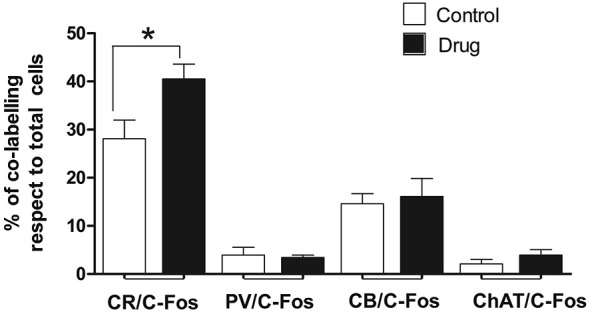
**Quantification of double immunofluorescence**. The number of double labeled neurons in the MS/VBD was expressed as percentage of total CR, CB, PV or ChAT positive neurons. Five to ten photographs were taken from at least three different subjects of control and 5 MPD groups.

To determine what kind of septal neurons were activated by acute MPD treatment, double immunofluorescence with c-fos antibody and different neuronal markers was carried out in both control and 5 MPD-treated rats. Quantification of at least 20 confocal images per sample of double staining from control and MPD treated rats, indicated that in basal conditions approximately 28.1 ± 3.9%, *n* = 4 of the CR-positive neurons co-labeled with c-fos and this percentage increased to 40.5 ± 3.1%, *n* = 4 after MPD treatment. On the other hand, the percentage of double labeling of c-fos with CB (14.6 ± 2.1, *n* = 3 basal; 16.1 ± 3.70, *n* = 4 MPD); PV (3.9 ± 1.7 basal; 3.4 ± 0.5, *n* = 4 MPD) and ChAT (2.1 ± 0.9%, *n* = 4 basal; 4.0 ± 1.1%, *n* = 4 MPD) did not change significantly with MPD treatment (Figure 4). Student *t-test*, *p* = 0.04. These results suggest that low doses of MPD targets mostly CR neurons in the MS/VDB area.

**Figure 5:**
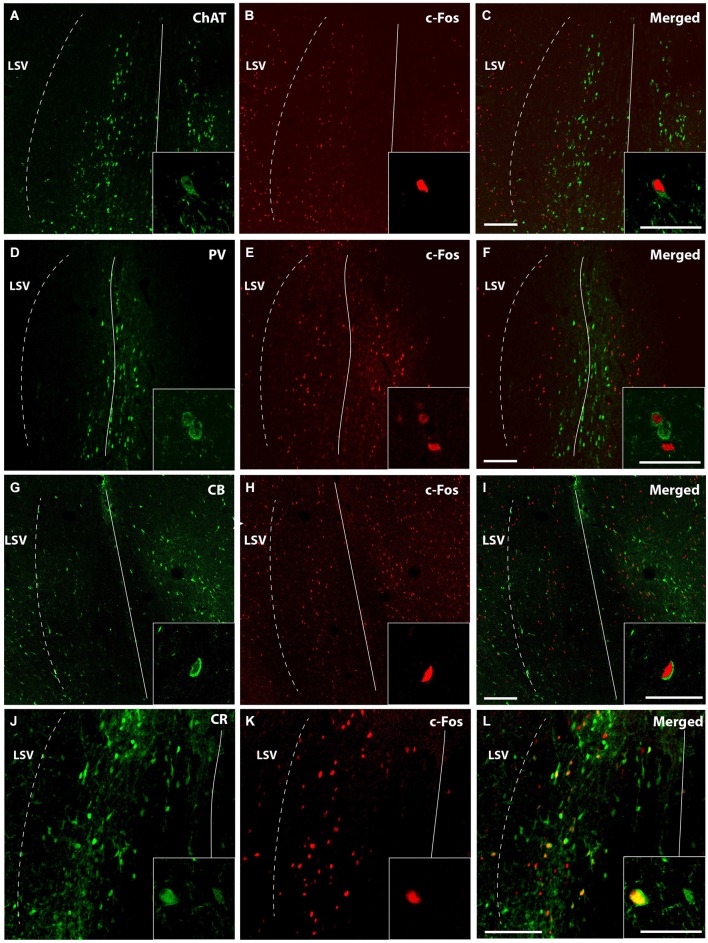
**Confocal images of double immunofluorescence**. Low magnification captures immunofluorescence of a representative case MPD 5 mg/Kg showing different areas occupied by ChAT (green) **(A)**; PV (green) **(D)**; CB **(G)**; and CR **(J)**. c-fos positive neurons (red) **(B,E,H,K)**; and merged **(C,F,I,L)**. Calibration bar 100 µm **(A–I)** and 50 µm **(J–L)**. High magnification of the double immunofluorescences was used for quantification and are represented in the insets. Calibration bar 50 µm.

Representative confocal immunofluorescence images of c-fos neurons from 5 MPD treated rats within the MS/VDB are shown (Figure 5). ChAT positive neurons (Figure 5A) occupy and area with some overlapping with c-fos positive neurons (Figure 5B), but little co-localization was observed (Figure 5C). Insets show the staining at higher magnification to demonstrate the labeling of single neurons. On the other hand, PV labeled neurons (Figure 5D) lay in central aspects of MS with little overlapping area with c-fos labeled cells (Figure 5E) and merged (Figure 5F). Similarly to ChAT neurons, CB (Figure 5G) and CR (Figure 5J) occupy more lateral aspects of the MS overlapping with c-fos positive area (Figures 5H,K). Representative images of merged photographs are shown (Figures 5I,L). High magnification representative images of co-labeled cells are shown in the insets.

## Discussion

In this paper we report an increase of c-fos expression specifically in calretinin neurons within the MS/VDB nuclei in the rat brain after MPD oral intake. MPD is a commonly prescribed drug for children with attention deficit disorder.

The drug doses and the pathway for drug administration are important factors to be taken into account when trying to understand physiological mechanisms of treatments used in human therapies studying animal models (Clark et al., 2007). Typically, given the existing differences in metabolism between rodents and humans, higher doses of different drugs (approx. 3-fold) are required to achieve blood levels in rats within the range found in humans (Gatley et al., 1999; Gerasimov et al., 2000). Children are typically treated with 0.25–1 mg/kg oral doses of MPD, yielding peak plasma MPD levels in the range of 8–40 ng/ml (Wargin et al., 1983; Swanson and Volkow, 2002). Studies in the adult rat showed that 0.5, 2, and 3.5 mg/kg oral administration results in peak plasma MPD concentrations of 2, 36, and 62 ng/ml, respectively (Aoyama et al., 1990). Similarly, Chase et al observed serum MPD levels of approximately 30, 150, and 390 ng/ml when administering 2.5, 5, and 10 mg/kg of oral MPD (Kuczenski and Segal, 2002; Bakhtiar and Tse, 2003; Chase et al., 2005). In addition, intraperitoneally or intraventricularly administered drugs are absorbed more rapidly and elicits stronger effects than oral treatments. Thus, MPD injected intraperitoneally (Schenk and Izenwasser, 2002) even at “clinically relevant” doses (2–5 mg/kg), yielded high serum levels (114–269 ng/ml), far exceeding clinical relevance (Gerasimov et al., 2000; Brandon et al., 2001; Schenk and Izenwasser, 2002).

Taken all this information together we considered the use of 1.3, 2.7, and 5 mg/Kg in order to be in the lower, but clinically relevant choice of MPD administration. Additionally, handling and administration through a plastic pipette mixture with jelly eliminate the stress produced by holding the rat and shouting with a needle. Previous studies have developed similar methods to orally administer drugs without the need of restraint, eliminating stress-induced c-fos activation that potentially could mask MPD-induced effects (Chase et al., 2005, 2007).

With this strategy, acute MPD (5 mg/kg; oral) has been reported to significantly increase extracellular dopamine levels in the nucleus accumbens and intensify locomotor activity (Chase et al., 2005). Moreover, it has been found that oral 7.5 mg/Kg MPD treatment augmented c-fos positive neurons in the striatum (Allen et al., 2010). Consistently, we observed an increase in c-fos expression within the striatum when administering 5 mg/Kg (but not at lower doses). Interestingly in our hands we found no effect within the nucleus accumbens. Furthermore, behavior effects have been reported with acute and orally given MPD (3 mg/Kg), improving learning and memory tasks in juvenile rats (Zhu et al., 2007). These results could be in accordance with our findings concerning c-fos expression in the MS/VDB when using 2.7 MPD. Lower doses MPD 2 mg/kg; (oral) has been proven to be ineffective (Gerasimov et al., 2000). Similarly, we found no changes when using 1.3 MPD.

MS has been mostly viewed as the source for subpallial input to the hippocampus (Risold, 2004). In addition, some tracing studies highlight the role of the MS as the source for descending projections targeting different hypothalamic and tegmental nuclei that, in turn, project back to the septum and hippocampus (Leranth et al., 1999; Sánchez-Pérez et al., 2015). On the other hand, the projections from the HDB target the amygdala, piriform cortex and olfactory bulbs (Swanson and Cowan, 1979). Thus, we could conclude that any alteration over the MS region will have effects in cognitive aspects rather than emotional ones, which would be more related to HDB activity.

Clinical effects of MPD are reported to improve awareness, in fact it is the current prescription for children with attention deficit disorders. Attention and awareness has been documented to depend on hippocampal rhythm, which is, in turn, modulated by septal-hippocampal circuitries (Bland and Oddie, 2001). A large body of evidence have demonstrated that the MS plays a central role in hippocampal function as measured by theta rhythm (Hajós et al., 2003, 2008; Krause et al., 2003; Berridge and Devilbiss, 2011). Moreover, manipulation of DA transmission in the septal area affects hippocampal theta synchronicity (Miura et al., 1987; Fitch et al., 2006), and a specific role for MS/VDB cells expressing D1/5 receptor in theta rhythm modulation has been observed (Fitch et al., 2006). Additionally, injection of D1 agonists in the MS has been reported to increase acetylcholine levels in the hippocampus (Zarrindast et al., 2012).

We have found that low levels of orally administered MPD increases neuronal activity, as measured by c-fos protein expression, specifically in the MS (at 2.7 mg/Kg doses) and at in the striatum (at 5 mg/Kg doses). c-fos has long been recognized as a marker for neuronal activation (Curran and Morgan, 1995; Kovács, 2008). Moreover, previous work had reported c-fos induction in different brain areas in response to high doses of intraperitoneally administered MPD (Trinh et al., 2003).

The MS/VDB is composed of different type of neurons; ChAT, PV, CB28kD and CR neurons (Kiss et al., 1997). ChAT and PV are hippocampal projecting cholinergic and GABAergic neurons (Freund, 1989). Only around 10% of CB28kD and CR neurons are GABAergic, while a major proportion, (40% approximately of CB28kD neurons and around 80% of CR neurons are glutamatergic (Gritti et al., 2003). It has been proposed that intrinsic septal glutamatergic projection may be involved in generation and modulation of hippocampal theta rhythm through projections to PV (Hajszan et al., 2004) and ChAT (Wu et al., 2004) septo-hippocampal neurons. In addition, CR neurons are the source of descending projections to hypothalamic (supramammillary) (Leranth et al., 1999) and tegmental (nucleus incertus) (Sánchez-Pérez et al., 2015) areas that are also involved in theta rhythm generation.

In order to elucidate the phenotype of neurons affected by MPD acute treatment, we labeled the cells with diverse neuronal markers and analyzed the double staining. We show that the percentage of CR positive neurons that co-localized with c-fos increased significantly after MPD treatment, indicating that in our conditions, MPD may target specifically CR neurons in the MS/VHD. Our data would be in accordance to the reported sensitivity of CR-GABAergic neurons to MPD treatment in the tail of the ventral tegmental area (VTA; Kaufling et al., 2010). Although there is no detailed description of DA receptors expression in CR septal neurons, striatal CR interneurons have been reported to express low to moderate levels of the D5 receptor (D1 subtype of DA receptors), whereas higher levels of D5 expression are found in PV and ChAT neurons (Rivera et al., 2002). On the other hand, β1 and β2 adrenergic receptors have been found in several subtypes of GABAergic, including CR, CB and PV, neurons in prefronal cortex (Liu et al., 2014). Interestingly, our current findings clearly support an action of low and clinical relevant doses of MPD specifically in CR neurons, whereas not affecting other subtypes of GABAergic neurons (PV, CB) nor cholinergic positive neurons within the MS/VDB.

Given the role of MPD as DAT inhibitor, this drug could be acting by increasing catecholamine transmission in the MS/VDB as it has been already reported in prefrontal cortex (Berridge et al., 2006). Interestingly, we observed few TH positive fibers inside the MS, and these results are consistent with other previously published (Lindvall and Stenevi, 1978; Fitch et al., 2006). However, DA neurotransmission can also occur by volume diffusion, meaning that it may not require synaptic proximity to elicit its action (Cragg et al., 2001). On the other hand, we cannot completely rule out a putative MPD effect on other neurotransmitters; for instance, MPD can induce histamine release in rat prefrontal cortex (Horner et al., 2007).

**Figure 6:**
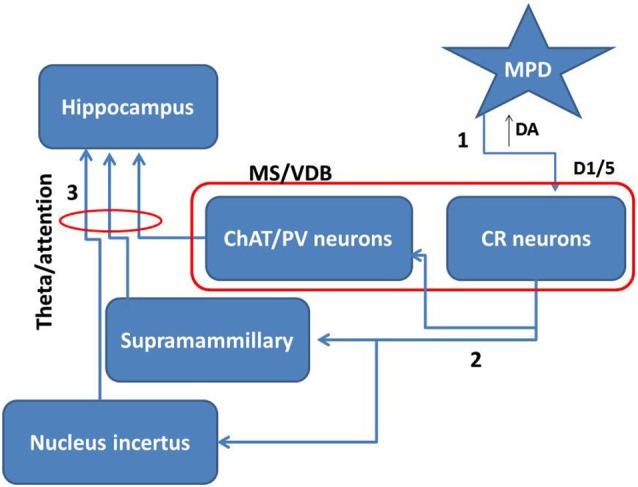
**Proposed mechanism for MPD role in attention**. Taking together the present data and others we propose that MPD may inhibit dopamine re-uptake leaving more dopamine in the extracellular space (1). This extra amount of dopamine will more effectively activate D1/5 receptors in the medial septal neurons. Specifically, the activation of CR neurons in this area will potentiate the effect on intrinsic connections inside the medial septum and descending projections over areas projecting to hippocampus (2). Then subcortical projections may enhance theta rhythm and consequently attention mechanisms (3).

Taking together all these data together with our own results, it is possible that MPD by inhibiting dopamine reuptake directly activates CR positive neurons within the MS/VDB. The increase of dopamine in the extracellular space may activate D1/5 receptors inducing potentiation of intrinsic GABAergic and descending glutamate projections to areas involved in theta activation (Figure 6). The action of MPD modulating awareness processes and supporting cognitive function is largely accepted, therefore, our data showing a specific effect of MPD on medial septal CR neurons, highlights the importance of this type of neurons in attention processes underlying hippocampal-dependent behavior.

## Conflict of Interest Statement

The authors declare that the research was conducted in the absence of any commercial or financial relationships that could be construed as a potential conflict of interest.
